# Surgical treatment of distal radius fractures: impact on forearm rotation in non-elderly patients

**DOI:** 10.1038/s41598-024-53520-3

**Published:** 2024-02-04

**Authors:** Lingde Kong, Chenfei Li, Jiangbo Bai, Jian Lu

**Affiliations:** Department of Orthopedics, Third Hospital of Hebei Medical University, Major Laboratory of Orthopaedic Biomechanics in Hebei Province, Shijiazhuang, 050051 Hebei China

**Keywords:** Trauma, Outcomes research

## Abstract

Forearm rotation restriction (FRR) is common after surgery for distal radius fractures (DRFs). The aim of the current study was to investigate the effect of DRFs on forearm rotation. This retrospective study reviewed patients with DRFs who underwent surgical treatment from January 2019 to December 2021. The patients' basic data and radiographic parameters were analyzed. Forearm rotation, including pronation and supination, was assessed using a standard goniometer. The Patient-Rated Wrist Evaluation (PRWE) score was evaluated, and the incidence of FRR at the 6-month follow-up was recorded. Univariate and multivariate logistic regression analyses were performed to identify risk factors correlated with FRR. A total of 127 patients with DRFs were included in this study. After surgery, 46 cases were considered to have FRR, with a rate of 36.2%, while the remaining 81 cases (63.8%) did not have FRR. The PRWE scores were 22.8 ± 5.2 and 17.9 ± 4.2 in the FRR group and non-FRR group, respectively, and the difference was statistically significant (*P* < 0.05). Multivariate analysis showed that the involvement of the sigmoid notch (OR, 2.88; 95% CI 1.49–5.56), post-operative volar tilt < 0° (OR, 2.16; 95% CI 1.34–3.50), and post-operative ulnar variance > 0 mm (OR, 1.37; 95% CI 1.06–1.78) were independently associated with the incidence of FRR. The FRR is associated with an increased PRWE score and may have had some impact on the patient's daily life. Fractures involving the sigmoid notch, dorsal angulation, and radial shortening deformity were found to be correlated with the incidence of FRR. Preoperative risk notification and intraoperative preventive measures are necessary for these patients.

## Introduction

Currently, distal radius fractures (DRFs) are the most common type of fractures in the upper limb, accounting for 20% of all fractures seen in emergency departments^1,2^. The mechanism of injury is usually a direct fall. Treatment strategies for DRFs vary, with closed reduction or surgery being effective options^3^. The primary goals of treatment are to achieve pain-free movement, allow early return to normal life, and minimize the future risk of disability or degenerative changes in the forearm. Surgical treatment is recommended for unstable or intraarticular DRFs as it provides good reduction and rigid internal fixation, allowing for better functional exercise and earlier return to normal activities. However, postoperative complications such as wrist pain, malunion, and restricted range of forearm motion can still occur^4,5^.

Normal forearm rotation involves complex movements, including rotation and dorsovolar translation of the distal radius on the distal radioulnar joint (DRUJ). This motion relies on the anatomical structure of the DRUJ, which includes the ulna, distal radius, and triangular fibrocartilage complex (TFCC)^6^. Coronal displacement affects DRUJ stability^7^. Soft tissue stabilizers include TFCC, pronator quadratus, ulnar carpal extensor tendon, DRUJ capsule, and interosseous membrane^8^. Distal interosseous membrane acts as a secondary stabilizer^9^. Residual ulnar translational deformity can impact DRUJ stability. DRFs can affect the DRUJ, leading to limited range of motion (ROM) in multiple planes^4^. Forearm rotation restriction (FRR) after DRFs may be caused by DRUJ subluxation and contracture of the surrounding soft tissues, including the joint capsule, volar or dorsal ligaments, and interosseous membrane. Despite FRR being common after DRFs, there have been limited studies investigating this issue, and the mechanism of rotational restriction in DRF patients is not clear. Therefore, we conducted this retrospective study to analyze factors associated with the incidence of FRR following DRFs treated with surgery. As the treatment strategy for DRFs differs between elderly and non-elderly patients^10–12^, and high-energy trauma often causes intra-articular or comminuted fractures in non-elderly patients with distal radius fractures. Surgical treatment, including internal fixation and plate osteosynthesis, is a popular option with new implant designs^13^. We conducted this research to provide assistance to clinical work for this specific target population.

## Materials and methods

### General information

This retrospective study reviewed non-elderly patients who underwent surgical treatment for DRFs between January 2019 and December 2021 in our institution. Our research has been approved by the Ethics Committee of the Third Hospital of Hebei Medical University (W2021-050-1), and all procedures were carried out in accordance with relevant guidelines and regulations, informed consent was obtained from all subjects and/or their legal guardian(s). The inclusion criteria were adults under 60 years old with closed DRFs confirmed by radiologic tests, who underwent volar locking plating and internal fixation (Shandong Weigao Medical Instrument Co., Ltd., Zibo, China). Patients with associated carpal bone fractures, open fractures, bilateral limb fractures, previous history of DRFs, or history of hand or forearm surgery were excluded. The study was approved by the Local Ethics Committee of our institution, and all patients provided written consent for storing their information in the institution's database for medical research purposes.

### Surgery and follow-up process

Surgeries were performed under brachial plexus or general anesthesia, with a pneumatic tourniquet applied to the upper arm. A standard volar approach between the flexor carpi radialis and radial artery was used to expose the fractures. After clear exposure of the distal radius, the reduction procedure was performed, followed by volar locking plating with or without K-wire fixation. Fixation was completed once satisfactory reduction was confirmed by intraoperative X-ray. After saline irrigation, the pronator quadratus was repaired, and the wound was closed.

All procedures were performed by three experienced surgeons. Internal fixation alone usually provides sufficient stability for early range of motion, but external fixation with short-arm plaster/splint may be used based on the surgeons' experience. Patients started shoulder, elbow, and finger exercises on the first day after surgery.

Follow-up visits were scheduled at 4, 6 and 8 weeks post-surgery. At each visit, X-ray tests were conducted to detect any early-stage problems. Once signs of fracture healing are observed (blurred fracture lines or formation of callus), the plaster/splint was removed, and range of motion exercises were initiated. At the 6-month follow-up, forearm rotation and functional assessment were recorded.

### Parameter assessment

**Figure 1:**
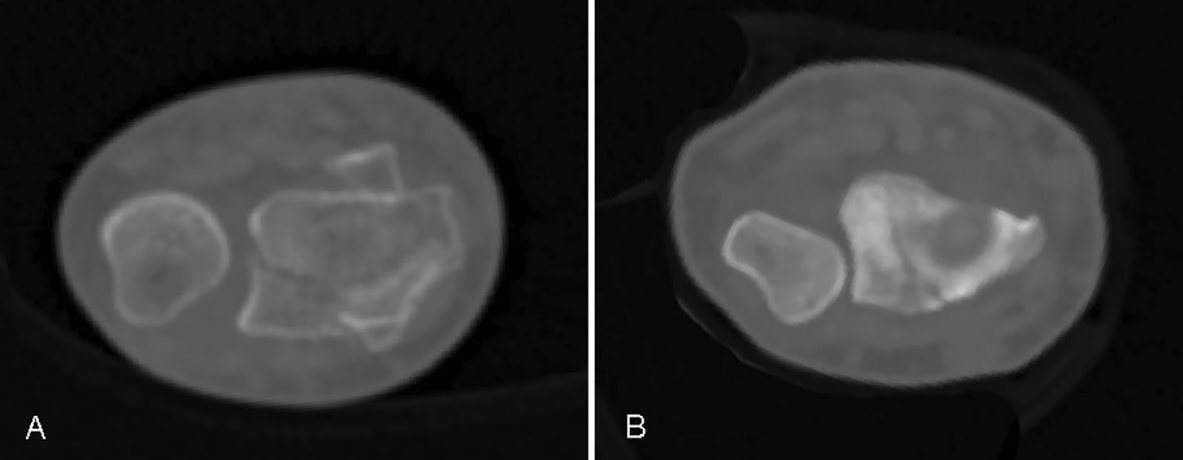
If fracture lines or steps were found on CT images at the site of the sigmoid notch, it was considered as positive (**A**) for involvement; otherwise, it was negative (**B**).

We collected patients' basic data from medical records, which included age, gender, sides of injury, and body mass index (BMI). Other variables were measured or evaluated. Swelling degree was assessed preoperatively. By conducting the “wrinkle test,” swelling was considered slight if the skin textures on the wrist could be recognized, and severe if the skin textures were not clear or blisters occurred^14^. Fracture type, involvement of sigmoid notch, and intactness of the ulnar styloid process were classified based on preoperative images. If fracture lines or steps were found on CT images at the site of the sigmoid notch, it was considered as positive for involvement; otherwise, it was negative (Fig. 1). Postoperative radiologic parameters were measured on X-ray photographs after the fractures had healed to avoid measurement inaccuracy caused by reduction loss. Radial inclination degrees, volar tilt degrees, and ulnar variance were three parameters used to assess radial inclination loss, dorsal angulation deformity, and radial shortening deformity, respectively, and were measured as previously described^4^. All imaging parameters in this study were performed by two radiologists with more than 10 years of clinical experience. The third observer intervened twice, to improve the accuracy and reliability of the measurement results. The attending surgeon provides personalized rehabilitation instructions to patients prior to discharge. These instructions are verbally communicated and repeated by the patient to ensure comprehension. Compliance with the instructions and the effectiveness of functional rehabilitation exercises are evaluated during follow-up visits, leading to categorization of patients into appropriate or inappropriate exercise groups. The ability of the wrist was evaluated using the Patient-Rated Wrist Evaluation (PRWE) system at the end of follow-up. While The PRWE is a simple, brief, reliable, and valid clinical tool using pain, and work performance as subjective and objective outcome indicators^15,16^. Forearm rotation, including forearm pronation and supination, was assessed using a standard goniometer. Forearm rotation restriction was defined as a range of pronation-supination movement that was less than two-thirds of that in the contralateral forearm.

### Statistical method

Continuous variables were presented as mean standard deviation, and categorical variables were presented as frequencies and percentages. The Fisher exact test was used for categorical variables, while the Mann–Whitney U test or the independent sample *t*-test was used for continuous variables to identify differences between groups. After univariate analyses, potential risk factors with a *P*-value less than 0.20 were entered into multivariate logistic regression models. The Statistical Package for the Social Sciences (SPSS, version 20.0) was used for all data analysis, and *P*-values less than 0.05 were considered statistically significant.

### Ethical approval

The research has been approved by the Ethics Committee of the Third Hospital of Hebei Medical University, and all procedures were carried out in accordance with relevant guidelines and regulations. We have no conflicts of interest to declare.

## Results

**Figure 2:**
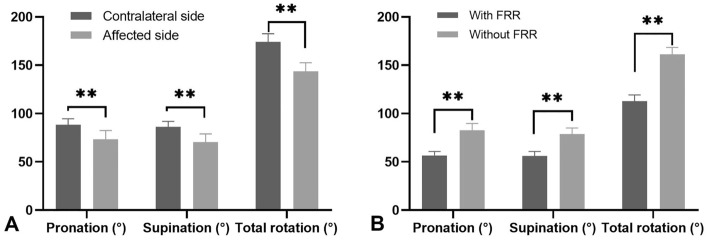
(**A**) The supination movement, pronation movement, and total rotation movement of the affected side were significantly worse than those of the contralateral side (*P* < 0.05). (**B**) The supination movement, pronation movement, and total rotation movement of these with FRR involved were significantly worse than those without FRR involved (*P* < 0.05). FRR: Forearm rotation restriction.

This study included a total of 127 patients with distal radius fractures (DRFs) with an average age of 44.0 ± 5.8 years. Among these patients, 52 (40.9%) were male and 75 (59.1%) were female. Fifty-nine injuries (46.5%) were on the left side, and the other 68 injuries (53.5%) were on the right side. At the 6-month follow-up, the pronation movement was 73.2° ± 9.2°, the supination movement was 70.4° ± 8.5°, and the total rotation range of motion (ROM) was 143.6 ± 8.9°. According to our criteria, 46 patients were considered to have forearm rotation restriction (FRR), with a rate of 36.2%, while the other 81 patients (63.8%) did not. The supination movement, pronation movement, and total rotation movement of the affected side were significantly worse than those of the contralateral side (*P* < 0.05). The detailed data of rotation movement were shown in Fig. 2. The PRWE score was 19.7 ± 4.5, and in the FRR group and non-FRR group, the PRWE scores were 22.8 ± 5.2 and 17.9 ± 4.2, respectively, with a statistically significant difference (*P* < 0.05, Table 1). In our observational cases, two patients presented with cutaneous infections at the site of Kirschner wire insertion. However, prompt removal of the wires, administration of oral antibiotics, and meticulous wound care effectively prevented any occurrence of deep-seated tissue infection. Both patients exhibited a favorable prognosis without any complications.

**Table 1:** Baseline data of DRFs patients.

Variable	Value
Total patients (no.)	127
Age (years), mean ± SD	44.0 ± 5.8
Gender	
Male (no.)	52
Female (no.)	75
Sides of injury	
Left (no.)	59
Right (no.)	68
Pronation (°), mean ± SD	73.2 ± 9.2
Supination (°), mean ± SD	70.4 ± 8.5
Rotation ROM (°), mean ± SD	143.6 ± 8.9
Patients with FRR (no.)	46
PRWE, mean ± SD	19.7 ± 4.5

DRFs, Distal radius fractures; SD, standard deviation; FRR, forearm rotation restriction; ROM, range of motion; PRWE, the patient-rated wrist evaluation system.

In the univariate analysis, we found that the number of FRR patients with fractures involving the sigmoid notch, and improper rehabilitation exercise was significantly higher than that in non-FRR patients. Additionally, the values of volar tilt and ulnar variance in FRR patients were significantly lower than those in non-FRR patients. Intra-articular fracture was a factor potentially associated with FRR (*P* = 0.06). The five potential risk factors with *P* < 0.2 in the univariate analysis will be included in the multivariable logistic regression model^17^, while age, gender, BMI, dominant hand, preoperative swelling, ulnar styloid process fracture, K-wire fixation, radial inclination, and the usage of cast or splint fixation were not (*P* ≥ 0.20). The detailed comparison between groups is listed in Table 2.

**Table 2:** The univariate analyses of potential risk factors associated with FRR.

Variables	With FRR (n = 46)	Without FRR (n = 81)	*P* value
Age (years)	43.2 ± 6.3	44.4 ± 5.6	0.27
Gender			
Male	17	35	0.58
Female	29	46	
BMI (kg/m^2^)	22.9 ± 1.9	23.1 ± 1.6	0.53
Dominant hand			
Yes	26	47	0.51
No	20	34	
Preoperative swelling			
Slight	18	38	0.46
Severe	28	43	
Fracture type			
Extra-articular	13	37	
Intra-articular	33	44	0.06
Involvement of sigmoid notch			
Yes	27	70	< 0.01
No	19	11	
Ulnar styloid process fracture			
Yes	19	38	0.58
No	27	43	
Internal fixation			
With K-wire	20	44	0.27
Without K-wire	26	37	
Radial inclination (°)	19.8 ± 2.4	19.2 ± 2.7	0.21
Volar tilt (°)	5.8 ± 1.3	6.9 ± 1.8	< 0.01
Ulnar variance (mm)	1.4 ± 0.3	− 2.1 ± 0.4	< 0.01
Usage of cast or splint fixation			
Yes	9	22	0.40
No	37	59	
Rehabilitation exercise			
Proper	20	53	0.03
Improper	26	28	

FRR, Forearm rotation restriction; BMI, body mass index.

In the further multivariate logistic regression analysis, involvement of the sigmoid notch (odds ratio [OR], 2.88; 95% confidence interval [CI], 1.495.56), postoperative volar tilt < 0° (OR, 2.16; 95% CI 1.34–3.50), and postoperative ulnar variance > 0 mm (OR, 1.37; 95% CI 1.06–1.78) were shown to be independently correlated with the incidence of FRR during follow-up (Table 3). Intra-articular fracture, postoperative volar tilt, or improper rehabilitation exercise were not considered risk factors.

**Table 3:** The multivariate logistic regression analysis of risk factors associated with FRR.

	*P* value	Odds ratio	95% CI
Intra-articular fracture	0.13	1.23	0.95–1.60
Involvement of sigmoid notch	0.01	2.88	1.49–5.56
Post-operative volar tilt < 0°	0.01	2.16	1.34–3.50
Post-operative ulnar variance > 0 mm	0.03	1.37	1.06–1.78
Improper rehabilitation exercise	0.07	1.45	0.99–2.12

FRR, Forearm rotation restriction; CI, confidence interval.

## Discussion

The radius rotates around the forearm rotational axis, passing from the center of the radial head to the ulnar fovea. The distal radius translates in the dorsal direction during supination and in the volar direction during pronation. This combined motion of rotation and translation of the distal radius relative to the ulna allows the forearm to achieve flexible movement^18^. As we know, compensation cannot be provided for forearm rotation via the shoulder or elbow, so restriction of forearm rotation is often disabling^19^. Clarifying the effect of DRFs on FRR can assist surgeons in identifying cases at a greater risk for dysfunction and modifying their treatment strategy in advance. In this study, we retrospectively reviewed patients who underwent surgery and revealed that the incidence of FRR was up to 36.2% during follow-up. The FRR is associated with an increased PRWE score and may have had some impact on the patient's daily life. Fractures involving the sigmoid notch, post-operative volar tilt of less than 0°, and post-operative ulnar variance of more than 0 mm were shown to be correlated with the incidence of FRR. Preoperative risk notification and intraoperative preventive measures are important for these cases.

The sigmoid notch is an essential part of forearm movement because it not only serves as an anchor for the TFCC, which plays an important role in wrist joint stability but also provides a smooth articular surface for rotational motion^20–22^. Our results showed that a fracture line involving the sigmoid notch is independently associated with FRR. A previous study by Kong et al. supported our results, reporting that DRFs involving the sigmoid notch had an adverse effect on forearm rotation after conservative treatment^23^. A reasonable explanation is that a displaced fracture may cause articular step-off, leading to articular incongruity, and ultimately limiting forearm rotation.

Radial inclination, volar tilt, and ulnar variance are three parameters frequently used to evaluate distal radius deformity and are considered to have a great influence on postoperative function^24,25^. Surgeons usually make every effort to achieve anatomical reduction; however, obtaining the anticipated reduction could be very difficult or even impossible for some patients. Nishiwaki et al. performed a biomechanical study in cadaveric specimens and confirmed that increasing volar angulation deformities of the distal radius decreased the range of forearm rotation^26^. Bessho et al. showed that volar angulation deformities increased the DRUJ stiffness in the intact TFCC^27^. Similar to these studies, we confirmed that dorsal angulation causes rotational restriction. We speculated that dorsal angulation could change the relationship between the radius and the ulna at the DRUJ and increase the tension of the TFCC, resulting in rotational dysfunction of the forearm.

Similarly, radial shortening was reported to reduce the amount of forearm rotation in a previous study^28^, and this conclusion was confirmed by our results. The specific reason may result from changes in TFCC tension in the same way. If so, combined dorsal angulation and radial shortening deformities may have a greater effect on forearm rotation. Although anatomic reduction is regarded as the ideal goal of surgical treatment, restoration of radial inclination is not demonstrated to be as critical as restoration of volar angulation or ulnar variance for forearm rotation movement. These results have important guiding significance for patients in the process of pursuing the recovery of forearm rotation function.

The strict inclusion criteria are an apparent advantage of our study. However, this study is limited by several points. Firstly, the retrospective study design and its potential bias in collected data are the main limitations of our study. Besides, Restricted by study design, limited risk factors included. More factors may add value. Potential risk factors found, exact reduction degree for reducing FRR not established. Thirdly, due to the lack of relevant imaging data, we were unable to evaluate the damage to TFCC and the reduction of the sigmoid notch after surgery. Finally, intra-articular fracture and improper rehabilitation exercise had been included as associated risk factors in the multivariable logistic regression model, but no statistical difference was shown after multivariate logistic regression analysis. This may result from the relatively small sample size, and further studies on this topic are still needed in the future.

## Conclusions

In summary, this study revealed that the incidence of FRR was up to 36.2% during follow-up. The FRR is associated with an increased PRWE score and may have had some impact on the patient's daily life. Fractures involving the sigmoid notch, dorsal angulation deformity, and radial shortening deformity were shown to be independently correlated with the incidence of FRR. Preoperative risk notification and intraoperative preventive measures are necessary for these patients.

## Data Availability

The data that support the findings of this study are available at reasonable request from the corresponding author.
